# The Effect of a Brief Video-Based Intervention to Improve AIDS Prevention in Older Men: Randomized Controlled Trial

**DOI:** 10.2196/65674

**Published:** 2025-04-18

**Authors:** Tian Huaju, Xie Rendie, Xiao Lu, Li Mei, Luo Yue, Zhang Daiying, Chen Yanhua, Ren Jianlan

**Affiliations:** 1 Department of the Operating Room, the Affiliated Hospital of Southwest Medical University, 25 Taiping Street, Luzhou, Sichuan Province, China; 2 Department of Anesthesiology, the Affiliated Hospital of Southwest Medical University, Luzhou, China; 3 School of Nursing, Southwest Medical University, Luzhou, China; 4 Department of Nursing, the Affiliated Hospital of Southwest Medical University, Luzhou, China

**Keywords:** older men, AIDS prevention, video, video-based intervention, HIV, AIDS

## Abstract

**Background:**

The AIDS epidemic among older people is becoming more serious. Evidence-based, acceptable, and effective preventive interventions are urgently needed. Video-based interventions have become an innovative way to change behaviors, and we have developed a brief video-based intervention named Sunset Without AIDS.

**Objective:**

In this study, we tested the effectiveness of a brief video-based intervention targeting older men’s understanding of AIDS prevention.

**Methods:**

A randomized controlled trial was conducted from June 20 to July 3, 2023. In total, 100 older men were randomly divided into the intervention group (n=50) and the control group (n=50) using the envelope extraction method. The intervention group was shown the Sunset Without AIDS video; the control group viewed a standard AIDS education video. A questionnaire was used to measure the effect of Sunset Without AIDS after 2 interventions. AIDS-related high-risk behaviors were followed up 1 and 3 months after the intervention. The difference was statistically significant at *P*≤.05.

**Results:**

After 2 interventions, the total awareness rates (%) of AIDS-related knowledge in the intervention and control groups were 84% (42/50) and 66% (33/50), respectively (*P=*.04). The mean stigma attitude scores of the 2 groups were 2.53 (SD 0.45) and 2.58 (SD 0.49), respectively (*P*=.55), but there was a statistically significant difference in the first dimension (fear of infection) between the 2 groups (*P*<.001). The mean positive scores of attitudes of AIDS-related high-risk behaviors of the 2 groups were 83.33 (SD 21.56) and 75.67 (SD 26.77), respectively (*P*=.58). In addition, 82% reported that they were satisfied with the educational content within the Sunset Without AIDS video. At 1- and 3-month follow-ups conducted after the intervention, participants in the 2 groups did not report AIDS-related high-risk behaviors. After watching the 2 videos, more people accepted and were satisfied with Sunset Without AIDS.

**Conclusions:**

Sunset Without AIDS could improve the ability of older men in China to follow best practices for AIDS prevention and provide a certain basis for the innovation of AIDS education in the older adult population.

## Introduction

With the acceleration of global population aging, AIDS has become increasingly prevalent among older adults, and the age composition of people living with HIV has gradually tilted toward older people. According to the Joint United Nations Programme on HIV/AIDS data [1], there were approximately 38 million people living with HIV worldwide and about 8.1 million of them were aged ≥50 years old. According to data released by the Chinese Center for Disease Control and Prevention (CDC) AIDS, the number of HIV-positive men over 60 years of age rose from 8391 in 2012 to 19,815 in 2017 [2] and 24,465 in 2018 [3].

On November 30, 2020, the Chinese CDC released core information on AIDS prevention. The core information has shown that in recent years, more than 95% of newly diagnosed people living with HIV in China contracted HIV through sexual means, and heterosexual transmission accounts for about 70% of these cases; unsafe sex is the main cause of sexual transmission of HIV [4]. The main route of HIV infection in older people in China is sexual transmission, and the imbalance of sexual demand is one of the causes of HIV infection in older people [5]. People remain sexually active after the age of 50, and high-risk sexual behaviors such as having multiple sexual partners and having unprotected sex with casual partners are common in the older population [6]. Due to insufficient AIDS-related knowledge or some personal factors, such as divorce, death of a spouse, or a spouse with no sexual needs, some older people, especially older men, may take part in unsafe sex under the influence of their friends and environment [5,7]. The survey found that up to 80% of older men with HIV admitted to having visited prostitutes, while older women were more likely to be infected within a marriage [7]. A survey showed that 94.7% of older men infected with HIV never used condoms when having sex with a commercial partner [8]. This shows that older men have a low degree of understanding of safe sex and do not take protective measures when having sex with strangers, so they are at risk of infection with and transmission of HIV [9].

Therefore, it is necessary to take effective measures to control the prevalence of HIV/AIDS among older men. Although there is no HIV vaccine available, other biomedical options, such as preexposure prophylaxis (PrEP) and postexposure prophylaxis, have had multiple challenges in terms of the rollout and implementation in China. In China, the key vulnerable population has insufficient awareness of these prevention methods and a low utilization rate. For example, only 20% of men who have sex with men were aware of PrEP and postexposure prophylaxis, and only <1% initiated PrEP [10,11].

Health education is an important tool in curbing the HIV/AIDS epidemic [12]. At present, AIDS health education targets mainly focus on adolescents [13-15] and men who have sex with men [16-18], with less focus on the older adult population. Furthermore, the existing AIDS awareness and education work is not accurate enough, and there is a lack of specific and strong popular science materials for older people [19]. At present, HIV/AIDS health education resources and methods for older adults are obsolete, and now awareness pamphlets, centralized lectures, publicity columns, and banners are more often used [20]. Older adults generally have poor eyesight, low levels of education, few ways of obtaining knowledge, and a lower ability to accept new knowledge. This makes it difficult for them to obtain correct HIV/AIDS prevention and control knowledge, so the effectiveness of education efforts is often not ideal [21,22]. In addition, there is limited discussion of the study’s global relevance beyond China [23,24]. Therefore, there is an urgent need to develop innovative models of AIDS education for older people and improve the educational effect, which can be feasibly implemented in overburdened health systems.

Video-based interventions are a promising but underused way to deliver AIDS education to older people. As a kind of teaching media, video interventions can deliver standardized information. At the same time, some useful information can be woven into engaging storylines, piloted to ensure cultural relevance, and delivered at critical teachable moments [25]. In addition to the aesthetic and entertainment value of such videos, they also contain great value in terms of health education.

In recent years, some randomized controlled trials were conducted in the United States and Africa targeting people living with HIV and high-risk populations. The trials found that video-based interventions were highly cost-effective [26] and demonstrated efficacy in improving health knowledge, supporting partner disclosure, increasing medication adherence, promoting HIV screening, and reducing sexual partners, as well as fostering attitude and behavior change [27-33]. However, due to regional and cultural differences, these video-based interventions do not conform to the national characteristics of HIV infection among older people in China. Meanwhile, these videos lack systematization, specialization, and guidance in the knowledge, attitude, and skills related to HIV infection among older people, so their promotion and application are limited. In China, there are few evidence-based studies on HIV/AIDS educational video interventions, especially randomized controlled trials to evaluate the effects of a video-based intervention on older men.

Considering the current HIV epidemic among older men in China, while recognizing that evidence-based, scalable, and accessible interventions to improve the ability of older men to prevent HIV are critical, we developed a brief video-based intervention (Sunset Without AIDS), mainly targeting education to increase AIDS-related knowledge, improve AIDS prevention motivation, and strengthen AIDS prevention behaviors among older men. The video is suitable for China’s national conditions and addresses the primary sexual issues related to HIV transmission among older people and can be promoted and applied in the older adult population.

In this study, we tested whether the Sunset Without AIDS video was effective in increasing AIDS-related knowledge, reducing AIDS-related stigma, and reducing AIDS-related high-risk behaviors among older men in Luzhou City, Southwest China. Here we report the results from this study.

## Methods

### Video Design

**Figure 1.**
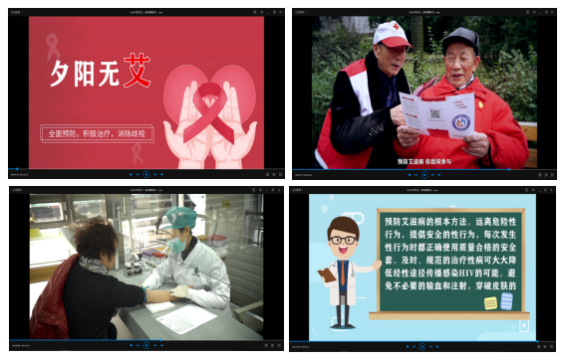
Screenshots of the Sunset Without AIDS intervention video.

The AIDS educational video used in this study is named Sunset Without AIDS and is 25 minutes long. In conceptualizing the video, the Information-Motivation-Behavioral (IMB) Skills Model was selected. The IMB Skills Model involves three aspects: AIDS-related information, motivation, and behavior skills [34]. It states that knowledge alone is insufficient for behavior change it must be linked to both motivation and behavioral skills to overcome barriers critical to achieving adherence [27]. Sunset Without AIDS incorporates all components of the IMB Skills Model and integrates other evidence-based video techniques to facilitate behavior change, such as video modeling and gain-framed information [35]. The gain-framed messaging emphasizes the advantages of behavior change, rather than the disadvantages of not changing behavior. It was selected to promote behavior change due to its demonstrated superiority over loss-framed messaging [36]. The script of Sunset Without AIDS is based on the true stories of older people living with HIV in China. The development of video content was through a community participatory approach, involving in-depth research and interviews with older men and people living with HIV to ensure that the educational content was acceptable and compelling. The script underwent iterative review and editing by a scriptwriter, until the expert advisory group felt the script addressed these issues in a compelling fashion. The characters in the video are all experienced actors and they speak Chinese. Sunset Without AIDS consists of two parts; one is about 18 minutes of story content, while the other is approximately 7 minutes of expert guidance [35]. In Sunset Without AIDS, the video provides information about the main route of transmission in the older population, the importance of HIV prevention, and education on HIV testing and treatment. Sunset Without AIDS is divided into multiple independent scenes in which topics including AIDS-related knowledge, attitude, and personal protection skills are interwoven into the dialogues and behavior attitudes of characters in specific plots. At the same time, the end of the story is supplemented with expert guidance, through expert authority, to increase awareness regarding HIV prevention and reduce high-risk behaviors related to HIV. The video covers three different health education themes: (1) facing sexual needs and guiding safe sex; (2) protecting yourself from HIV; (3) facing the disease bravely and preventing AIDS scientifically [25]. Screenshots of Sunset Without AIDS are shown in Figure 1.

### Study Design

A parallel design randomized controlled trial was conducted. The sample size calculation was based on the primary outcome, that is, AIDS-related knowledge. Sufficient statistical power to detect changes in AIDS-related knowledge after the intervention was considered in the sample size calculation, and 36 participants were required in each group to achieve 80% statistical power. The study took place in Kuangchang Town, Luzhou City, which is a Chinese administrative division containing 11 communities. To facilitate the implementation of the trial, a simple random sampling method was used to select 3 communities in Kuangchang Town. Based on the size of the older population in these 3 communities, a total of 100 older men who met the requirements of the trial were recruited in the 3 communities using quota sampling through community advertising and on-site recruitment. Research assistants delivered talks to all eligible participants describing the trial. When the participants agreed to join the study and completed the follow-up measurement, an informed consent form needed to be signed for confirmation. Once participants’ eligibility was determined, they were scheduled to complete a baseline survey. The baseline survey included demographic information, AIDS-related knowledge, stigma attitude, AIDS-related attitudes to high-risk behaviors, and AIDS-related high-risk behaviors.

To reduce contamination, the trial took place at 2 different community centers. For the study, 100 participants were randomly assigned to 1 of the 2 intervention groups on a 1:1 basis. Opaque envelopes were prepared for randomization. Each of the 100 envelopes contained a note with the words “Sunset Without AIDS intervention group” or “Normal video group” (50 envelopes per group). Participants (n=100) were randomly assigned a computer number from 1 to 100, and they randomly selected an envelope in numbered order. For each envelope selected by participants, the total number of envelopes was reduced by 1 until the end. Participants who selected the envelopes in turn opened them in front of a third-party staff member and were assigned to different groups based on the information on the envelope. Another staff member recorded the allocation information of the participants.

After 2 interventions, all participants were evaluated for their AIDS-related knowledge, stigma attitude, and AIDS-related high-risk behaviors attitude. Meanwhile, face-to-face interviews were used to evaluate the feelings of all participants in the intervention group toward Sunset Without AIDS. The two groups’ AIDS-related high-risk behaviors were followed up 1 and 3 months after the intervention. Three months after the intervention, the convenience sampling method was used to select 10 older men from the intervention group. After watching the videos of the intervention group and the control group, the 10 older men were interviewed face-to-face to evaluate their satisfaction with and acceptance of the intervention.

We had the following hypotheses: (1) Sunset Without AIDS can increase the awareness of AIDS-related knowledge, reduce AIDS-related stigma, and reduce HIV-related high-risk behaviors among older men; and (2) in a comparison between the Sunset Without AIDS intervention group and the normal video group, the Sunset Without AIDS intervention group would integrate various knowledge and behavioral skills into the story. So, the Sunset Without AIDS intervention could have better acceptability and feasibility for older men’s education.

### Participants

We recruited 100 older men in three communities in Kuangchang Town, Luzhou City in Southwest China through community advertising and on-site recruitment. The eligibility criteria for participation were as follows: (1) male aged over 60 years old; (2) clear consciousness, no mental illness and serious visual impairment, has a certain level of reading ability and language expression ability; and (3) plans to stay in the trial catchment area for over 3 months. The exclusion criteria were as follows: (1) ongoing or already participating in an AIDS education intervention; and (2) cognitive and mental impairment, inability to communicate properly, or blurred vision or blindness.

### Intervention

The intervention group and control group received the corresponding intervention from June 20 to July 3, 2023. The intervention group received 25 minutes of the Sunset Without AIDS video once per week for 2 weeks. During the viewing, a member of the study team was responsible for answering questions. To ensure the compliance of participants, each participant could receive recognition and a reward of 50 RMB (US $6.91) in cash after completing each video viewing and follow-up assessment.

Participants in the normal video group received an AIDS education video intervention downloaded from the website of the Chinese CDC, which was composed of 5 short video clips, about 25 minutes in total. Intervention times and duration were the same as the Sunset Without AIDS group. The video contained similar educational content as the Sunset Without AIDS, including AIDS-related knowledge and attitude and behavior education. The only difference is that it is a popular science informational video, which lacks a storyline and is not specifically aimed at older people. As in the intervention group, a member of the study team was responsible for answering questions from the participants during the viewing.

### Outcomes and Measurements

The primary outcomes comprised change in score, that is, increased AIDS-related knowledge, decreased AIDS-related stigma attitude, and improved AIDS-related high-risk behaviors attitude. The secondary outcomes were the feelings of the intervention group after 2 interventions and their AIDS-related high-risk behaviors 1 and 3 months after the intervention. At the same time, the satisfaction and acceptance of both interventions were also evaluated after 3 months of intervention.

AIDS-related knowledge was assessed through 8 core knowledge questions on AIDS commonly used by the Chinese AIDS Prevention and Control Committee [19]. One point was given for a correct answer. The higher the score, the more knowledge participants have obtained. The total awareness rate of AIDS knowledge refers to the proportion of the respondents who correctly answered 6 or more of the 8 basic AIDS knowledge items simultaneously.

AIDS-related stigma attitude was assessed through the Chinese version of Zelaya et al’s [37] HIV/AIDS Stigma Scale. The scale was translated into Chinese by Xing et al in 2014 [38]. All items are divided into four dimensions: fear of infection, stigma prejudice, personal discrimination, and social discrimination, with 6 items for each dimension, for a total of 24 items. The scale contains 6 positive items and 18 negative items, and the answers are strongly agree, agree, uncertain, oppose, and strongly disagree. Items are scored on a 5-point scale (1 to 5) and negative items are reverse scored. The higher the participants’ discrimination score, the more severe the AIDS discrimination [39].

AIDS-related high-risk behaviors attitude was assessed through a 6-item questionnaire. It included attitude toward AIDS-related high-risk behaviors such as seeking HIV testing and counseling, seeking sexual partners through commercial/social/internet methods, having one-night stands, having multiple sexual partners, and having unprotected sex. The higher the questionnaire score, the lower the participant’s risk of engaging in AIDS-related high-risk behaviors. The content validity of the questionnaire was evaluated by 5 experts; the item-level content validity index and the scale-level convent validity index were both 1.00.

AIDS-related high-risk behaviors were assessed through a self-made questionnaire with 3 items [22]. The questionnaire included whether someone sought sexual partners through business, social, or online means, and whether they had sex with strangers. The fewer AIDS-related high-risk behaviors the participants had, the better their ability to prevent HIV/AIDS.

The outline of the user experience interview mainly included: (1) whether you accepted the education of AIDS related-knowledge in the form of watching videos; (2) were you satisfied with the educational content of the Sunset Without AIDS video; (3) whether the story and theme of the video could attract you and arouse your attention; (4) after watching the video, whether you had a clear understanding of the main route of HIV infection in older people, and emphasized the use of condoms; and (5) did you know how and where to get tested for HIV after watching the video?

### Data Analysis

SPSS (version 21.0; IBM Corp) statistical software was used for data analysis. Percentage was used for statistical descriptions of classified data, and *χ*^2^ test was used for qualitative data to compare each indicator; the statistical description of the quantitative data was given using mean (SD), while *t* test was used to compare each indicator between groups, and *P*≤.05 was considered a statistically significant difference. The data analysts were blinded to the allocation.

### Ethical Considerations

Study approval was gained from the ethics review committees of the Affiliated Hospital of Southwest Medical University (number KY2021071). This study was registered at the Chinese Clinical Trial Registry (ChiCTR2100045708). All participants who participated in the survey signed an informed consent form. Each participant could receive 50 RMB (US $6.91) in cash after completing each video viewing and follow-up assessment. The informed consent form also included a statement that study data were anonymous or would be deidentified and could be used in a secondary analysis of data without additional consent.

## Results

**Figure 2.**
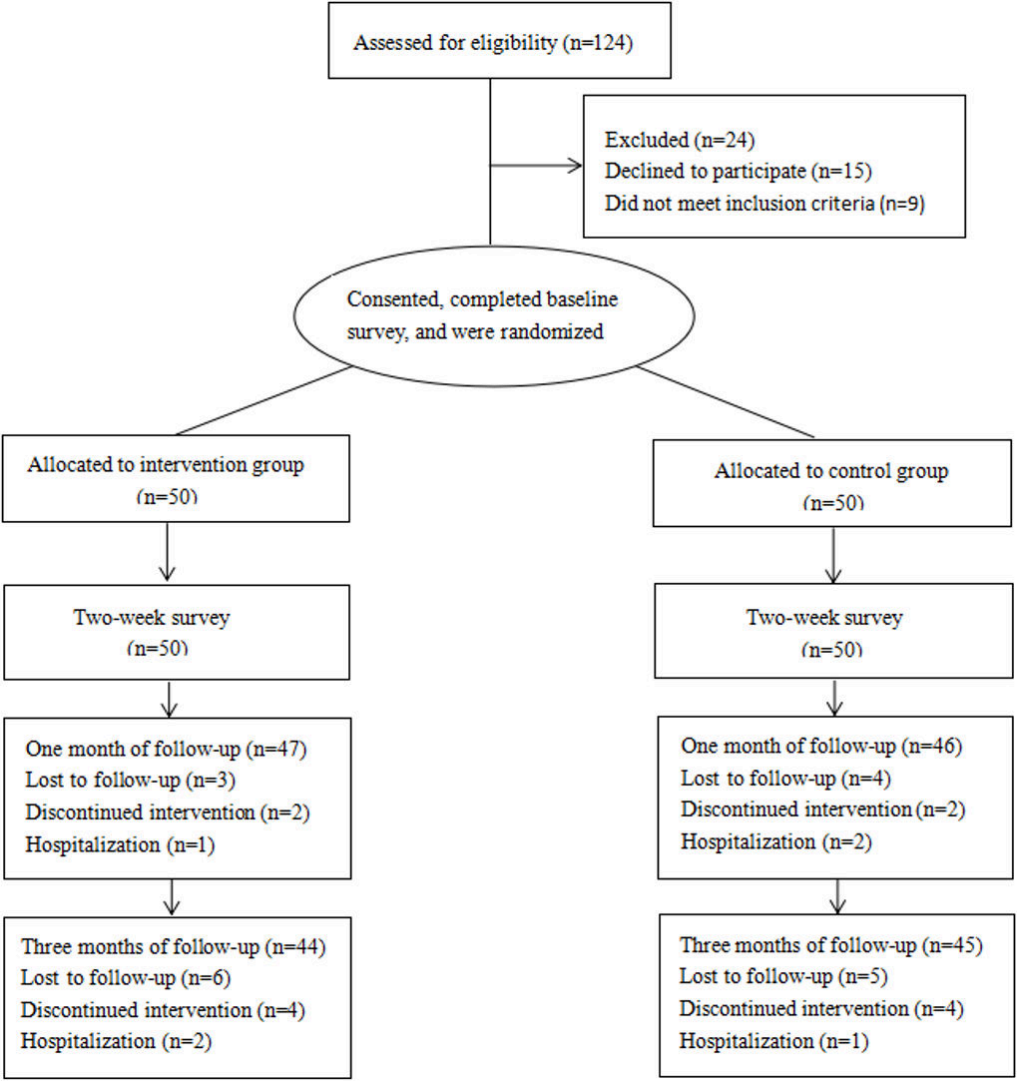
The participant flow throughout the study from screening to follow-up.

Figure 2 depicts participant flow throughout the study from screening to follow-up.

### Description of Study Sample

The study sample included 100 participants (intervention group: n=50, control group: n=50) aged between 60 and 78 years old, all Han nationality. Urban older people accounted for 72% (n=72) of the sample, and rural older people accounted for 28% (n=28). Married, divorced, and widowed people accounted for 77% (n=77), 8% (n=8), and 15% (n=15), respectively. Illiteracy, primary school, junior high school, and academic education accounted for 8% (n=8), 33% (n=33), 34% (n=34), and 25% (n=25), respectively. Participants were mainly from enterprise institutions (n=30, 30%) and farming (n=27, 27%). The 2 groups of participants acquired AIDS-related knowledge mainly through television and hearing it from others. There were no significant demographic differences between the 2 groups, with comparability (Table 1).

**Table 1.** Participant demographics (n=100).

Characteristics		Intervention group (n=50)	Control group (n=50)	*t* test/chi-square (*df=98*)	*P* value
Age (years), mean (SD)		68.62 (4.72)	68.48 (4.99)	0.129	.90
**Marriage, n (%)**				0.684	.71
	Married	37 (74)	40 (80)		
	Divorced	5 (10)	3 (6)		
	Widowed	8 (16)	7 (14)		
**Education, n (％)**				1.25	.74
	Illiterate	3 (6)	5 (10)		
	Primary school	15 (30)	18 (36)		
	Junior high school	18 (36)	16 (32)		
	Academic	14 (28)	11 (22)		
**Occupation, n (％)**				0.477	.98
	Enterprise institution	14 (28)	16 (32)		
	Business	3 (6)	2 (4)		
	Farming	14 (28)	13 (26)		
	No job	9 (18)	8 (16)		
	Other	10 (20)	11 (22)		
**Inhabiting information, n (％)**				0.88	.83
	Solitary	10 (20)	9 (18)		
	Couple (partner) living together	10 (20)	14 (28)		
	Live with children	18 (36)	16 (32)		
	Three or more generations live together	12 (24)	11 (22)		
**Access to AIDS information (multiple choices available), n (％)**					
	TV	44 (88)	42 (84)	0.332	.56
	Newspaper	14 (28)	8 (16)	2.098	.15
	Internet	19 (38)	12 (24)	2.291	.13
	Broadcast	12 (24)	15 (30)	0.457	.50
	Community and hospital publicity	13 (26)	10 (20)	0.508	.48
	Hearing	35 (70)	40 (80)	1.333	.25
	Other	2 (4)	5 (10)	1.382	.24

### Comparison of Indicators Between the Two Groups Before the Intervention

Two people in the intervention group reported seeking sexual partners through business, social networking, or the internet and having sex with strangers. Both reported not using condoms when having sex with strangers. Three people in the control group reported seeking sexual partners through business, social networking, or the internet and having sex with strangers. Among them, 2 reported not using condoms when having sex with strangers, and 1 reported sometimes using condoms when having sex with strangers. Before the intervention, there was no statistically significant difference in the AIDS-related knowledge, stigma attitude, AIDS-related high-risk behaviors attitude, and AIDS-related high-risk behaviors between the 2 groups (Table 2).

**Table 2.** Comparison of indicators between the two groups before intervention (n=100).

Items	Intervention group (n=50)	Control group (n=50)	*t* test/chi-square (*df=98*)	*P* value
**Total awareness rate of AIDS-related knowledge, correct rate, n (%)**	15 (30)	20 (40)	1.099	.30
1. Is it possible to get AIDS if you share syringes with a person with HIV?	37 (74)	32 (64)	1.169	.28
2. Can HIV infection be seen on the outside?	32 (64)	33 (66)	0.044	.83
3. Are children born to women with HIV likely to develop AIDS?	26 (52)	31 (62)	1.02	.31
4. Can proper condom use reduce HIV transmission?	36 (72)	40 (80)	0.877	.35
5. Can mosquito bites spread HIV?	8 (16)	13 (26)	1.507	.22
6. Can you get AIDS from eating with people with HIV or sick people?	27 (54)	29 (58)	0.162	.69
7. Does having sex with only one partner reduce HIV transmission risk?	35 (70)	32 (64)	0.407	.52
8. Can you get AIDS from HIV-infected blood?	36 (72)	37 (74)	0.051	.82
**Stigma score, mean (SD)**	2.91 (0.38)	2.99 (0.52)	0.897	.37
Dimension 1: fear of infection	3.16 (0.57)	3.22 (0.74)	0.482	.63
Dimension 2: stigma prejudice	3.12 (0.52)	3.12 (0.70)	0.05	.96
Dimension 3: personal discrimination	2.48 (0.47)	2.46 (0.53)	0.13	.90
Dimension 4: social discrimination	2.89 (0.77)	3.16 (0.69)	1.898	.06
**Positive attitude rate of AIDS-related high-risk behaviors, %, mean (SD)**	58.33 (32.34)	59.33 (32.67)	0.15	.88
1. If you have high-risk behavior, will you take the initiative to seek HIV testing and counseling?	54.00 (50.35)	56.00 (50.14)	0.216	.83
2. Do you agree with the behavior of seeking sexual partners through business, social networking, or the internet?	62.00 (49.03)	66.00 (47.85)	0.389	.70
3. Do you approve of having a one-night stand with a stranger?	72.00 (45.36)	70.00 (46.29)	0.24	.81
4. Do you approve of having multiple sexual partners?	72.00 (45.36)	70.00 (46.29)	0.227	.82
5. Do you agree to use condoms if you have sex with strangers?	46.00 (50.35)	48.00 (50.47)	0.24	.81
6. Do you approve of taking Viagra and other drugs during sex?	44.00 (50.14)	46.00 (50.35)	0.184	.86

### Comparison of Indicators Between the Two Groups Before and After the Intervention

After 2 interventions, the total awareness rate of AIDS-related knowledge in the intervention group was higher than that in the control group (*P*<.05). The difference in the stigma score was not statistically significant after the intervention (*P*=.55). However, there was a statistical difference in the first dimension (fear of infection) between the 2 groups (*P*<.001). The difference in the positive attitude rate of AIDS-related high-risk behaviors was also not statistically significant after the intervention (*P*=.12). Before and after the intervention, the total awareness rate of AIDS-related knowledge and positive attitude rate of high-risk AIDS-related behaviors in both groups were higher than before the intervention, and the stigma score was lower than before the intervention; the difference was statistically significant (*P*<.01; Table 3).

**Table 3.** Comparison of indicators between the 2 groups before and after 2 interventions.

Items	Intervention group (n=50)				Control group (n=50)				Difference between the 2 groups after the intervention	
	Before intervention	After intervention	*t* test/chi-square (*df=98*)	*P* value	Before intervention	After intervention	*t* test/chi-square (*df=98*)	*P* value	*t* test/chi-square (*df=98*)	*P* value
**Total awareness rate of AIDS-related knowledge, correct rate, n (%)**	15 (30)	42 (84)	29.743	<.001	20 (40)	33 (66)	6.784	.01	4.32	.04
1. Is it possible to get AIDS if you share syringes with a person with HIV?	37 (74)	47 (94)	7.44	.006	32 (64)	43 (86)	6.453	.01	1.778	.18
2. Can HIV infection be seen on the outside?	32 (64)	40 (80)	3.175	.08	33 (66)	32 (64)	0.044	.83	3.175	.08
3. Are children born to women with HIV likely to develop AIDS?	26 (52)	46 (92)	19.841	<.001	31 (62)	43 (86)	7.484	.006	0.919	.34
4. Can proper condom use reduce HIV transmission?	36 (72)	46 (92)	6.775	.009	40 (80)	41 (82)	0.065	.78	2.21	.14
5. Can mosquito bites spread HIV?	8 (16)	29 (58)	18.919	<.001	13 (26)	17 (34)	0.762	.38	5.797	.02
6. Can you get AIDS from eating with people with HIV or sick people?	27 (54)	40 (80)	7.644	.006	29 (58)	35 (70)	1.563	.21	1.333	.25
7. Does having sex with only one partner reduce HIV transmission risk?	35 (70)	43 (86)	3.73	.053	32 (64)	41 (82)	4.11	.04	0.298	.58
8. Can you get AIDS from HIV-infected blood?	36 (72)	47 (94)	8.575	.003	37 (74)	46 (92)	5.741	.02	0.154	.70
**Stigma score, mean (SD)**	2.91 (0.38)	2.53 (0.45)	6.20	<.001	2.99 (0.52)	2.58 (0.49)	9.531	<.001	0.604	.55
Dimension 1: fear of infection	3.16 (0.57)	2.69 (0.67)	5.86	<.001	3.22 (0.74)	2.79 (0.76)	5.488	<.001	3.921	<.001
Dimension 2: stigma prejudice	3.12 (0.52)	2.80 (0.61)	3.662	.001	3.12 (0.70)	2.71 (0.69)	6.779	<.001	0.69	.49
Dimension 3: personal discrimination	2.48 (0.47)	2.14 (0.45)	4.975	<.001	2.46 (0.53)	2.27 (0.59)	3.023	.004	1.375	.18
Dimension 4: social discrimination	2.89 (0.77)	2.50 (0.64)	4.18	<.001	3.16 (0.69)	2.57 (0.52)	7.382	<.001	0.628	.53
**Positive attitude rate of AIDS-related high-risk behaviors, %, mean (SD)**	58.33 (32.34)	83.33 (21.56)	5.935	<.001	59.33 (32.67)	75.67 (26.77)	4.085	<.001	1.592	.12
1. If you have high-risk behaviors, will you take the initiative to seek HIV testing and counseling?	54.00 (50.35)	88.00 (32.83)	4.629	<.001	56.00 (50.14)	82.00 (38.81)	3.487	.001	0.829	.41
2. Do you agree with the behavior of seeking sexual partners through business, social networking, or the internet?	62.00 (49.03)	92.00 (27.41)	4.20	<.001	66.00 (47.85)	86.00 (35.05)	3.13	.003	1.00	.32
3. Do you approve of having a one-night stand with a stranger?	72.00 (45.36)	92.00 (27.41)	3.13	.003	70.00 (46.29)	92.00 (27.41)	3.348	.002	0.00	>.99
4. Do you approve of having multiple sexual partners?	72.00 (45.36)	88.00 (32.83)	2.419	.02	70.00 (46.29)	86.00 (35.05)	2.682	.01	0.299	.77
5. Do you agree to use condoms if you have sex with strangers?	46.00 (50.35)	74.00 (44.31)	4.365	<.001	48.00 (50.47)	60.00 (49.49)	1.52	.14	1.549	.13
6. Do you approve of taking Viagra and other drugs during sex?	44.00 (50.14)	66.00 (47.85)	2.852	.006	46.00 (50.35)	48.00 (50.47)	0.216	.83	1.843	.07

### Evaluation of User Experience in the Intervention Group

After 2 interventions, the user feelings of the intervention group after watching the Sunset Without AIDS video were evaluated by face-to-face interview. Overall, 92% (46/50) reported that they were willing to accept AIDS-related knowledge education in the form of watching videos and 82% (41/50) reported that they were satisfied with the educational content of the Sunset Without AIDS video. In addition, 94% (47/50) reported that the story and theme of the video could attract them and arouse their attention and 90% (45/50) reported that after watching the video, they had a clear understanding of the main route of HIV infection in older people and emphasized the use of condoms. Finally, 88% (44/50) reported that they knew how and where to conduct HIV testing after watching the video. The specific feelings of the intervention group after watching the video mainly focused on the 3 aspects displayed in Textbox 1.

Textbox 1. **The specific feelings of the intervention group.**

**AIDS awareness**

1. Some understanding about HIV/AIDS.

2. Know how HIV is spread.

3. AIDS had been thought to be a disease of the young, but it turned out to be a disease of older people as well.

**Personal and social behavior**

1. Be responsible for our family, be responsible for our children.

2. We should not discriminate against people with AIDS.

3. The sexual needs of older people should be faced squarely.

4. Society should strengthen the propaganda and guidance of AIDS prevention.

5. Pay attention to safe sex and use condoms.

**Evaluation of the Sunset Without AIDS intervention**

1. The video was very meaningful it felt like the people and things in the video were happening around us.

2. It was closer to our life for older people.

3. The story of the video was very good and could attract me very much.

4. This way was very suitable for older adults.

### Follow-Up Evaluation of AIDS-Related High-Risk Behaviors

After 1 and 3 months of the intervention, the 2 groups were followed up to evaluate the changes to AIDS-related high-risk behaviors. The follow-up rates of the intervention group and the control group were 94% (47/50) and 92% (46/50) 1 month after the intervention. Three months after the intervention, the follow-up rates of the intervention group and control group were 88% (44/50) and 90% (45/50), respectively. At 2 follow-up visits, participants in both groups reported that they did not seek sexual partners through business, social networking, or the internet and did not have sexual activities with strangers after the intervention. The main characteristics of sexual behavior were compared between those who were lost to follow-up and those who had participated in the follow-up evaluation. The results showed that before the intervention, none of those lost to follow-up reported seeking sexual partners through business, social networking, or the internet and engaging in sexual activities with strangers. Compared with before the intervention, AIDS-related high-risk behaviors decreased in the 2 groups, but there was no statistical significance (*P*>.05).

### Evaluation of Acceptance and Satisfaction

After 3 months of follow-up, 10 older men in the intervention group were interviewed after watching the videos of the intervention group and the control group. The results showed that, compared with traditional forms of health education such as AIDS book publicity and lectures, 7 reported that they were more willing to watch videos, 2 reported that they were more willing to use traditional health education, and 1 reported that there was no difference. After watching the Sunset Without AIDS video and normal AIDS education video, 9 accepted and were satisfied with the Sunset Without AIDS video.

## Discussion

### Principal Findings

In this study, a randomized controlled trial was conducted to evaluate the effectiveness of Sunset Without AIDS on preventing HIV among older men in China. The results showed that before the intervention, 100 participants had a very low awareness rate of AIDS-related knowledge and a serious discriminatory attitude toward AIDS, which was basically consistent with the relevant survey results [22,40-43]. Among AIDS-related knowledge, for the question “Can mosquito bites spread HIV?” only 21% were correct. It showed that there was a certain misunderstanding of the method of HIV transmission and a lack of understanding of AIDS-related knowledge among the older men. This suggested that it is urgent to carry out effective AIDS health education for older people.

The study found that after the intervention, the total awareness rate and item 5 awareness rate of AIDS-related knowledge in the intervention group were higher than those in the control group; the difference was statistically significant (*P*<.05). It was suggested that the education effect of the Sunset Without AIDS video was better than that of the normal AIDS propaganda video in improving the knowledge level of AIDS in older men. At present, HIV/AIDS prevention and treatment focused on scientific and accurate propaganda content, but ignored the relevance of the propaganda content for the target population [44]. Sunset Without AIDS considered and told the story from the perspective of older people, which was not only more targeted, but also in line with the daily life of older people. In the form of a film, Sunset Without AIDS vividly showed the experience of HIV infection among older people, which conveyed educational information, and greatly attracted users’ interest in learning and enhanced their learning motivation. At the same time, participants had access to targeted expert guidance to strengthen the relevant knowledge to improve the effect of education.

Studies have shown that all film and television materials have the potential to profoundly affect emotions and knowledge [45]; this was consistent with the results of this study. After the intervention, the total awareness rate of AIDS-related knowledge in both groups was higher than that before the intervention (*P*<.01). This result was consistent with the application effect of video-based intervention in college students [45]. After the application of the Sunset Without AIDS intervention, the total awareness rate of AIDS-related knowledge in the intervention group and the awareness rate of AIDS-related knowledge in the other items except item 2 and item 7 were all higher than before the intervention and the differences were statistically significant. Among them, the awareness rate of item 3 and item 5 increased most obviously (*P*<.001). This showed that Sunset Without AIDS had a good effect on the propaganda and popularization of nonsexual transmission of AIDS under the educational background that focused on sexual transmission of AIDS.

Influenced by traditional concepts in China, older adults generally avoid talking about sexual issues, and this group had greater discrimination and prejudice against HIV/AIDS than other age groups [19]. Sunset Without AIDS showed some effect in reducing AIDS**-**related stigma among older men; it has the potential to become an effective educational tool to reduce AIDS**-**related stigma among older men. The study found that although there was no statistically significant difference in the AIDS**-**related stigma attitude score between the 2 groups after the intervention, there was a statistical difference in the first dimension (fear of infection*; P*<.001). This might be related to the positive emotion emphasized in Sunset Without AIDS, and the portrayal of optimistic and strong images presented by the media. On the one hand, it uses sympathy and empathy to make the viewer think and consider themselves in that position, helping them understand the plight of people living with HIV to reduce discrimination against them. On the other hand, the intervention depicts the brave face of people living with HIV while HIV/AIDS prevention volunteers answer the older adults’ questions, underscoring that normal daily life communication will not lead to the spread of HIV/AIDS so as to ease public concern.

Attitude has a very important effect on the occurrence of behavior. At present, interventions targeted at AIDS-related stigma reduction are mainly in the form of peer education, health lectures, and face-to-face counseling. After intervention, a high positive rate of AIDS-related stigma attitudes can be maintained in the short term, but the long-term effect is not good or needs to be studied [46-48]. This suggests that in changing AIDS-related stigma in older adults, we need to adopt some better, more sustainable and effective intervention tools and intervention methods.

The results showed that after 2 interventions, the total positive attitude rate of AIDS-related high-risk behaviors and the positive rate of each item in the intervention group were increased; the improvement degree was statistically significant (*P*<.05). Among them, items 1, 2, and 5 showed the most significant improvement (*P*<.001). This shows that Sunset Without AIDS could improve the positive attitude rate of AIDS-related high-risk behaviors in older men. This might be because the video introduced the core knowledge of AIDS in an easy-to-understand way while adding some warning educational content. Through watching this content, the older men could realize the severe situation of the AIDS epidemic and enhance their awareness of self-protection [49], thus promoting a positive change in AIDS-related high-risk behaviors attitude.

The user experience evaluation results of Sunset Without AIDS suggested that the users had a high overall evaluation of this video, especially in the evaluation of “whether the story and theme of the video could attract them and arouse their attention”—as high as 94% of the older men said yes. In terms of the evaluation of users’ specific feelings, they were particularly satisfied with the story and immersion of the film. It showed that in terms of design, the film with story combined the scientific aspects of AIDS education with the entertainment value of a film, which could fully attract and mobilize the interest of users and satisfy their curiosity and thirst for knowledge. It was worth noting that in the interviews with users, some older men put forward that: “The sexual needs of older people should be faced squarely.” Compared with previous studies, Sunset Without AIDS did not blindly emphasize abstinence, but directly faced the primary sexual issue involved in the spread of AIDS among older people.

Follow-up data found that, although there was a reduction in AIDS-related high-risk behaviors attitude between the two groups, the difference was not statistically significant compared with before the intervention. The reasons might be as follows: (1) the sample size of this study was small, which might lead to insignificant differences; and (2) the intervention was only carried out twice and the follow-up survey of AIDS**-**related high-risk behaviors was only 1 and 3 months after the intervention. If a long-term intervention could be conducted and the follow-up time could be extended to evaluate the long-term effects, significant differences might be obtained.

The study found that there was no significant difference in some educational effects between the Sunset Without AIDS intervention and the normal video intervention, but compared with the normal video intervention, having one with a storyline that integrated various knowledge and behavioral skills led to better acceptance and satisfaction and it was more favored by the older men. Therefore, as a potential, targeted, and innovative AIDS health education tool, Sunset Without AIDS is worth sharing and promoting among older men.

### Limitations

This study is a pilot randomized controlled trial and the total sample size was small (100 cases). In addition, the intervention time was short—only 2 weeks. The participants of this study were only recruited in Kuangchang Town, Luzhou City. It did not include research participants from different cities and regions, so the representativeness of the research participants might be insufficient. Among the samples, the urban older adults accounted for a high proportion, and most of them were between 60 and 70 years old, which might result in the overrepresentation of this group. In the next study, we will conduct a large-scale, continuous, multicenter, gender-specific study to improve the scientific nature of the study.

HIV/AIDS and sex are both relatively private and sensitive topics; therefore, in Sunset Without AIDS, there may be some educational content that the older men cannot accept. In the future, we will improve the content of the video, integrate AIDS education into topics that the older men are interested in, and make the educational content more simple and easier to understand to have a greater effect on AIDS-related knowledge, stigma, and sexual behavior.

In addition, the next step will also be to evaluate the long-term effect of the Sunset Without AIDS video and study the mechanism of action, laying the foundation for the large-scale promotion and application of this video.

### Conclusion

Sunset Without AIDS could improve the ability of older men in China to prevent AIDS and provide a certain basis for the innovation of AIDS education in the older adult population. At the same time, it could also have a broader impact on public health policy formulation and practice. It could effectively increase AIDS-related knowledge among older men, but there was no observed effect in reducing AIDS-related stigma, improving attitudes toward high-risk AIDS behaviors, and reducing AIDS-related high-risk behaviors. Large-scale and long-term effects studies are needed to assess the efficacy of the Sunset Without AIDS intervention.

## Supplementary material

10.2196/65674 Checklist 1 CONSORT-EHEALTH V1.6.

## Abbreviations

CDC: Center for Disease Control and Prevention
IMB: Information-Motivation-Behavioral
PrEP: preexposure prophylaxis
